# Efficient Photodynamic Therapy against Gram-Positive and Gram-Negative Bacteria Using THPTS, a Cationic Photosensitizer Excited by Infrared Wavelength

**DOI:** 10.1371/journal.pone.0011674

**Published:** 2010-07-20

**Authors:** Stanislaw Schastak, Svitlana Ziganshyna, Burkhard Gitter, Peter Wiedemann, Thomas Claudepierre

**Affiliations:** 1 Department of Ophthalmology, Medical Faculty, University of Leipzig, Leipzig, Germany; 2 Department of Anaesthesia and Intensive Care Medicine, Medical Faculty, University of Leipzig, Leipzig, Germany; 3 Research & Development, Biolitec AG, Jena, Germany; Tufts University, United States of America

## Abstract

The worldwide rise in the rates of antibiotic resistance of bacteria underlines the need for alternative antibacterial agents. A promising approach to kill antibiotic-resistant bacteria uses light in combination with a photosensitizer to induce a phototoxic reaction. Concentrations of 1, 10 and 100µM of tetrahydroporphyrin-tetratosylat (THPTS) and different incubation times (30, 90 and 180min) were used to measure photodynamic efficiency against two Gram-positive strains of *S.aureus* (MSSA and MRSA), and two Gram-negative strains of *E.coli* and *P.aeruginosa*. We found that phototoxicity of the drug is independent of the antibiotic resistance pattern when incubated in PBS for the investigated strains. Also, an incubation with 100µM THPTS followed by illumination, yielded a 6lg (≥99.999%) decrease in the viable numbers of all bacteria strains tested, indicating that the THPTS drug has a high degree of photodynamic inactivation. We then modulated incubation time, photosensitizer concentration and monitored the effect of serum on the THPTS activity. In doing so, we established the conditions to obtain the strongest bactericidal effect. Our results suggest that this new and highly pure synthetic compound should improve the efficiency of photodynamic therapy against multiresistant bacteria and has a significant potential for clinical applications in the treatment of nosocomial infections.

## Introduction

For the past 60 years, antimicrobial chemotherapy has been the mainstay of medical intervention against infectious diseases caused by bacterial pathogens. The continuous decline of therapeutic effectiveness, as a result of extensive use of antibiotics, has long been predicted [1] and many surveillance efforts over the last decade have drawn attention to this phenomenon that now imposes a large burden on health care facilities [2]–[5].

Since its first appearance in 1960 [6], methicillin resistant *Staphylococcus aureus* (MRSA) has become widespread in hospitals and intensive care units (ICUs) [7], and now accounts for >60% of *S.aureus* isolates in US hospital and ICUs [8]. MRSA infections kill ∼19,000 hospitalized American patients annually; equivalent to the combined number of deaths due to AIDS, tuberculosis, and viral hepatitis [9]. Appearance of new resistance against vancomycin further aggravate the problem [10], [11]. Last but not least, the increase of Gram-negative pathogens, such as *Escherichia coli* (*E. coli*) and *Pseudomonas aeruginosa* (*P .aeruginosa*), with resistance to all antimicrobial drugs [12], [13] has also stimulated an extensive search for alternative antimicrobial treatment, especially for localized infections of the skin and oral cavity.

Antimicrobial photodynamic therapy (PDT) was described more than one hundred years ago by O. Raab and H. von Tappeiner. It was completely neglected during the golden age of antibiotic but it is slowly moving out of limbo to offer new therapeutic opportunities against multiresisitant bacteria [14]–[16]. Photodynamic inactivation of microorganisms is based on the properties of dyes, known as photosensitizers, to be preferentially localized in the bacteria and not in the surrounding tissue. They are subsequently activated by low doses of visible light of an appropriate wavelength, generating free radicals or singlet oxygen that are toxic to target microorganisms [17], [18]. Unlike antibiotics, repeated photosensitizations of bacteria do not induce the selection of resistant strains as singlet oxygen and free radicals interact with several cell structures and different metabolic pathways in microbial cells [19].

In the past ten years, the use of Photodynamic treatment against MRSA has dramatically increased [20]–[23]. The limitation of this technique is linked to photosensitizer properties, including the amount of energy needed to activate the photosensitizer [24], [25], low penetration depth of laser light due to its activating wavelength [24]–[26], the charge and purity of the molecule [25]–[29], specificity of the photosensitizer for bacteria [30] and the uptake kinetic of the compound in microorganisms [31]. Therefore, the positively charged and water-soluble tetrahydroporphyrin dyes have considerable advantages in comparison to other photosensitizers. They have strong absorption bands in the infrared region and show much higher uptake in negatively charged mitochondria membrane, which are key regulators of apoptosis by activating the caspase cascade [32], [33].

We previously demonstrated that the tetracationic photosensitizer tetrahydroporphyrin-tetratosylat (THPTS) has a stronger bactericidal effect on Gram-positive bacteria, in both methicillin sensitive *S. aureus* (MSSA) and MRSA, compared to Photolon, a dianionic chlorine e6 sodium salt drug that has been approved for photodynamic therapy. We also demonstrated its efficiency against Gram-negative bacteria (*S.aeruginosa and E.coli*) while anionic Photolon was inefficient [34]. In the present study we further studied the bactericidal efficiency of THPTS on Gram-positive (MSSA and MRSA) and Gram-negative (*E.coli* and *P.aeruginosa*) bacteria. We determined the optimal incubation times for all bacterial strains tested and demonstrated that THPTS can mediate antimicrobial effect of PDT at lower concentration than previously shown. In addition, for MRSA/MSSA and *E.coli*, the bactericidal effect of THPTS was also observed in presence of serum protein, thus matching more closely the condition of patient treatment.

Altogether our results suggest that this new and highly pure synthetic compound should improve the efficiency of PDT against multiresistant bacteria with a significant potential for clinical applications in the treatment of nosocomial infections.

## Results

### Viability of Gram-positive bacteria

**Figure 1:**
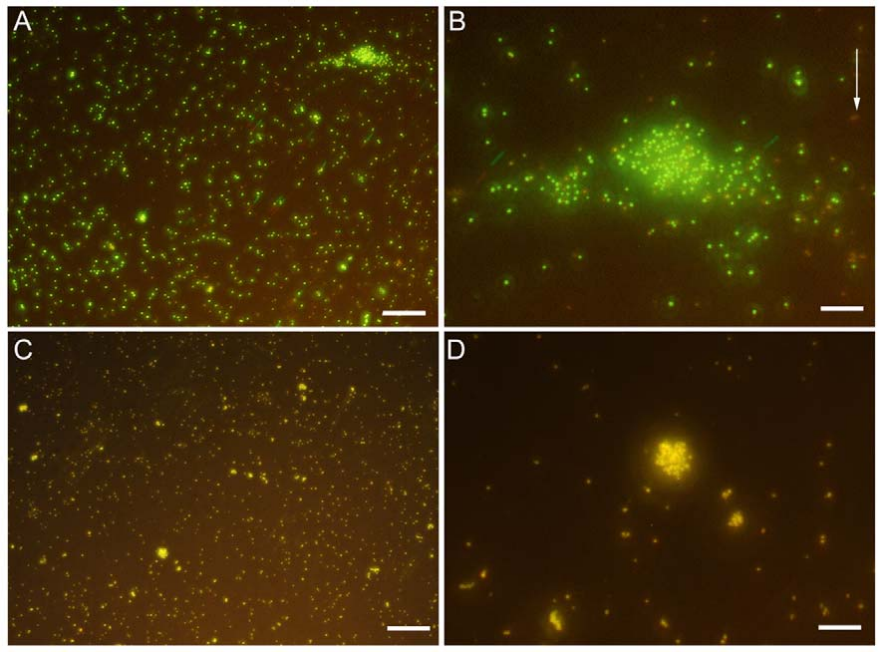
Viability of Gram-positive bacteria using a live/dead assay. Cells were incubate for 90min with THPTS and either kept in the dark (A, B) or irradiated (C, D) at 760nm. Healthy bacteria were seen in green whereas dying bacteria were also stained with propidium iodie and appeared therefore in yellow. Dying bacteria could be observed in control panels (arrows in B) but no healthy bacteria were seen in irradiated panel (C and D). Scale bar 40µm in A and C, 10µm in B and D.

MSSA were stained using Syto9 to stain the nuclei and propidium iodide to detect damaged DNA, causing healthy cell to be stained in green whereas dying bacteria appear yellow (Fig. 1). In the control situation (Fig 1 A and magnification in B), when incubated with THPTS but without being exposed to proper excitation wavelenght for the photosensitizer, bacteria appeared labelled in green only, thus demonstrating that THPTS has no toxicity per se. Few bacteria appeared labelled in yellow (arrows) and account for the normal rate of dying cells in the suspension. After incubation with THPTS and excitation, all bacteria were labelled with propidium iodide and also appeared then in yellow (Fig 1 C and magnification in D). Therefore, damages induced by THPTS were due to the photodynamic reaction and not to an intrinsinc toxicity of THPTS. Before examination, bacteria were kept in the dark for only 15 min following laser excitation; therefore, generation of free radicals and ROS took place rapidly after illumination at proper wavelenght.

### Photoinactivation of methicillin-sensitive S.aureus (strain: DSM 1104)

**Figure 2:**
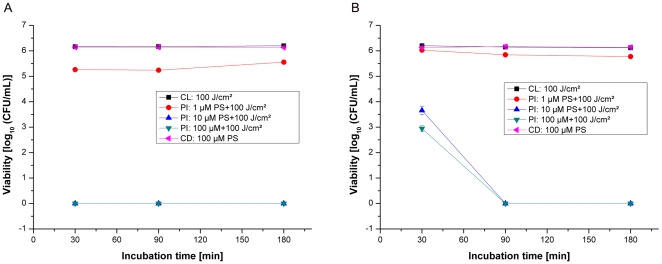
Photoinactivation of MSSA by THPTS. Photoinactivation of *S.aureus* (MSSA strain: DSM1104) by THPTS after incubation times of 30, 90 or 180min in PBS (A) or in PBS+10% HS (human serum, B). Viability of MSSA was determined by a CFU assay. Even in presence of serum, 10µM of THPTS for 90min had a dramatic bactericidal effect leading to the total absence of colony. Each point is the mean ± standard deviation of three experiments (P<0.005). CD: control in the dark, bacteria were maintained in the dark after incubation with THPTS. CL: control with light, no THPTS incubation before laser treatment. PI: photoinactivation using THPTS at indicated concentrations before laser treatment.

Without photosensitizer treatment, all bacterial samples exhibited normal growth, demonstrating that the maximal irradiation dose of 100J/cm^2^ alone had no antibacterial effects. Also, without any irradiation, incubation of bacterial samples for 30; 90 and 180min with different concentrations (1, 10 and 100µM) of THPTS did not induce any killing effect, thus confirming the photodynamic mechanism of action. Only a small bactericidal effect of nearly 0.5log_10_ (colony-forming unit/ml, CFU/mL) was measured after irradiation following all incubation times of bacteria with THPTS in PBS at a concentration of 1µM (Fig. 2). At this concentration, THPTS effect cannot be exacerbated by increasing the incubation time. An irradiation of MSSA bacteria, incubated with THPTS at concentrations of 10 or 100µM in PBS for 30, 90 or 180 min shows significant (P<0.005) antibacterial activity of more than 6log_10_ (CFU/mL) for all the incubation time tested.

In order to match closer the condition of patient treatment we then analyzed the effect of human serum (HS) addition (10% in PBS). In this condition, 30min of incubation using 10 and 100µM THPTS caused only a moderate decrease of about 2log_10_ (CFU/mL) after illumination. It is clear that PDT efficiency can be dramatically perturbed by the addition of sera. However, increasing the incubation times to 90 or even 180 minutes allowed us to overcome the consequence of serum addition and to obtain again a total absence of bacterial colony in PBS+10%HS using 10 and 100µM of THPTS. In this bacterial strain and in the presence of serum, increasing the incubation time is sufficient to recover the complete bactericidal effect of THPTS at 10 and 100µM . Even in presence of higher human serum concentrations, the photobactericidal effect remained complete with 25% HS using 10 and 100µM THPTS and above 2log_10_ for the highest concentration of THPTS in the presence of 50 and 100% HS (CFU/mL, see supplemental data, Fig. S1).

### Phototoxicity against methicillin-resistant S.aureus (strain: DSM 11729)

**Figure 3:**
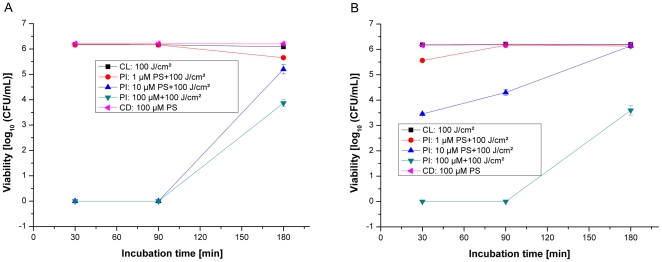
Photoinactivation of MRSA by THPTS. Photoinactivation of *S.aureus* (MRSA strain: *DSM11729*) by THPTS after incubation times of 30, 90 and 180min in PBS (A) and in PBS+10% HS (B). Viability of MRSA was determined by a CFU assay. 10µM of THPTS for 30min were sufficient to obtain a total absence of colony. However this concentration had to be increased to 100µM in presence of serum. Each bar is the mean ± standard deviation of three experiments (P<0.005). CD: control in the dark, CL: control with light, PI: photoinactivation.

In order to investigate whether the observed growth reduction of MSSA was independent of the antibiotic resistance pattern, a MRSA strain was photosensitized under identical conditions to those used for the MSSA strain. Without being photosensitized all MRSA samples exhibited normal growth with or without illumination, demonstrating that the maximal irradiation dose of 100J/cm^2^ alone had no antibacterial effects. At the concentration of 1µM THPTS no photokilling effect was observed for any period of incubation in PBS as well as in PBS+10% HS. However for higher THPTS concentrations, MRSA strain shows similar decreases as MSSA in CFU/ml after incubation and irradiation (Fig. 2). Irradiation of the MRSA in the presence of 10 and 100µM of THPTS in PBS resulted in a complete absence of CFU per millilitre (Fig. 3). The total photokilling of the MRSA of more than 6log_10_ (CFU/mL) was reached with 30 and 90min incubations with 10µM and 100µM of THPTS in PBS and with 100µM THPTS in PBS+10% HS. Using 10µM of photosensitizer in 10% HS the photobactericidal effect decreased but still remained at 3log_10_ (CFU/mL). Here we observed that incubation time has to be limited to 90 min for optimal effect of THPTS and that serum does not dramatically influence the efficiency of the photosensitizer when used at 100µM. Using an incubation time of 90min in PBS as well as in PBS+10% HS, a photokilling efficacy of more than 6log_10_ (CFU/mL) was always obtained with 100µM THPTS. A further increasing of incubation time to 3 hours induced a decrease in the killing rate of these bacteria, both in PBS and in presence of serum, a phenomena probably linked to the pharmacokinetic of the compound in this specific bacterial strain.

### Viability of Gram-negative bacteria

**Figure 4:**
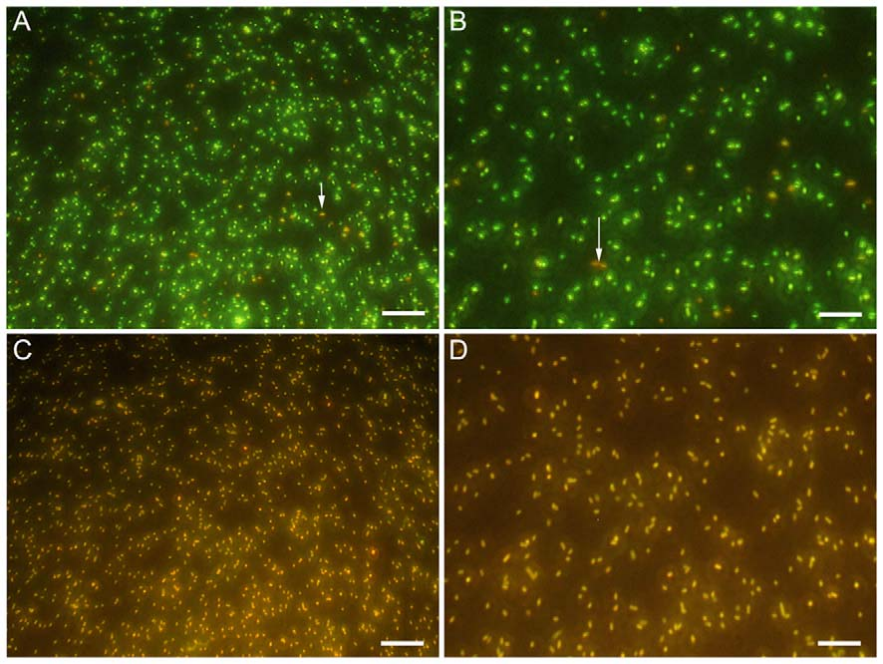
Viability of Gram-negative bacteria using live/dead assay. Cells were incubate with THPTS for 90min and either kept in the dark (A, B) or irradiated (C, D) at 760nm. Healthy bacteria were seen in green whereas dying bacteria were also stained with propidium iodide and appeared therefore in yellow. Dying bacteria could be observed in controls (arrows in A and B) but no healthy bacteria were seen in irradiated samples (C and D). Scale bar 40µm in A and C, 10µm in B and D.

*E.coli* were stained using Syto9 to stain the nuclei and propidium iodide to detect damaged DNA, causing healthy cell to be stained in green whereas dying bacteria appear yellow (Fig. 4). In the control situation, (Fig 4A and magnification in 4B) when incubated with THPTS but without being exposed to proper excitation wavelength for the photosensitizer, only few *E.coli* were seen labelled in yellow (arrows in Fig. 4A and B). This accounts for the normal rate of dying cells in the suspension. After incubation with THPTS and excitation, all bacteria were labelled with propidium iodide and then also appeared in yellow (Fig 4C and magnification in 4D). Therefore, damages induced in *E.coli* by THPTS were due to the photodynamic reaction and not to an intrinsic toxicity of THPTS. Before examination, bacteria were kept in the dark for only 15min following laser excitation; therefore, generation of free radicals and ROS took place rapidly after illumination at 760 nm.

### Phototoxicity against E.coli (strain: DSM8698)

**Figure 5:**
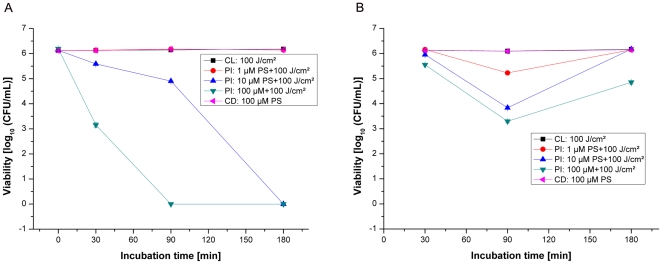
Photoinactivation of *E. Coli* by THPTS. Photoinactivation of *E. coli (DSM8698)* by THPTS photosensitizer (PS) after incubation times of 30; 90 and 180min in PBS (A) or in PBS+10% HS (B). Viability of bacterial cells was determined by a CFU assay. 100µM of THPTS for 90min, or 10µM for 180min were sufficient to obtain an efficient bactericidal effect. However, serum addition reduced the efficiency of the treatment without abolishing it when THPTS was incubated for 90min. Each bar is the mean ± standard deviation of three experiments (P<0.005). CD: control in the dark, CL: control with light, PI: photoinactivation.

The incubation of *E.coli* in the dark with THPTS showed no decrease in the numbers of CFU per millilitre. Irradiation of the Gram-negative bacterium *E.coli* after incubation with THPTS at concentrations identical to those in the experiments mentioned above revealed only a small decrease in the log_10_ (CFU/mL) numbers compared with staphylococcal strains (Fig. 2 to 3). When incubated for 30min with THPTS in PBS+10% HS, little or none photobactericidal effect was observed at all mentioned concentrations (Fig. 5). Irradiation of *E.coli* incubated for 90min with the concentration of 10µM of THPTS in PBS and in PBS+10% HS resulted in a moderate decrease in the numbers of CFU per millilitre of around 1 to 2log_10_ (CFU/mL). A longer incubation time of 180min in PBS, using 10µM of THPTS, showed a significant increase in the killing rate of *E.coli* in comparison to the 90min incubation time. However this effect was abolished in the presence of human serum. A moderate photobactericidal effect of around 3log_10_ (CFU/mL) was measured after incubation with 100µM of THPTS for 30min in PBS. Nevertheless, the incubation in PBS at the same THPTS concentration of 100µM induced a total killing effect of more than 6log_10_ (CFU/mL) when incubation time was extend to 90 and 180min. Notably, this bactericidal effect using 100µM of THPTS was diminished in the presence of human serum to but not negated.

### Phototoxicity against *P.aeruginosa* (strain: DSM1117)

**Figure 6:**
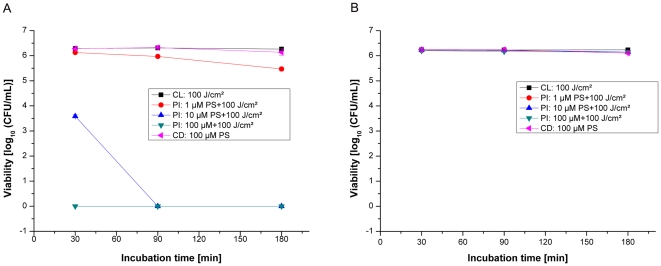
Photoinactivation of *P.aeruginosa* by THPTS. Photoinactivation of *P.aeruginosa (DSM1117)* by THPTS photosensitizer (PS) after incubation times of 30, 90 and 180min in PBS (A) and PBS+10% HS (B). 100µM of THPTS for 30min or 10µM of THPTS for 90min were sufficient to obtain a total bactericidal effect leading to the complete absence of colony. Serum addition completely abolished the bactericidal effect of THPTS for this bacterial strain. Viability of bacterial cells was determined by a CFU assay. Each bar is the mean ± standard deviation of three experiments (P<0.005). CD: control in the dark, CL: control with light, PI: photoinactivation.

In the dark, the incubation of *P.aeruginosa* with THPTS at the highest tested concentration of 100µM showed no decrease in the numbers of CFU per millilitre, after 30, 90 and 180min incubation times in PBS and PBS+10% HS (Fig. 6 A and B). Also, no photobactericidal effect was observed after the incubation of *P.aeruginosa* with THPTS at a concentration of 1µM for 30min in PBS and for incubations with THPTS at all concentrations and incubation times in PBS+10% HS. A moderate photobactericidal effect of around 2.5log_10_ (CFU/mL) was measured after incubation with a dose of 10µM of THPTS for 30min in PBS. The photobactericidal effect was increased by extending the incubation period to 90min. A total killing of *P.aeruginosa* of more than 6log_10_ (CFU/mL) was measured after incubation with 100µM THPTS for 30, 90 and 180min incubation times in PBS.

## Discussion

### Bactericidal properties of THPTS

We demonstrated that the PDT inactivation of methicillin-sensitive and methicillin-resistant strains of *S.aureus* with THPTS showed a clear THPTS dose-dependent viability loss after irradiation even after incubation in PBS+10% HS (Figs. 1 and 2). It should be noted that in the presence of different concentrations of human serum, the growth delay of Gram-positive MRSA bacteria was nearly a linear function of its concentration.

Different results were found for Gram-negative bacteria. We observed a high rate of photoinactivation in *E.coli* that was preincubated for 90 or 180min with THPTS at 10 or 100µM in PBS. A moderate photoinactivation of 2.5 and 3log_10_ (CFU/mL) was observed for PBS+10% HS after 90min incubation of the bacteria with THPTS at concentrations of 10 and 100µM, respectively. A high photoinactivation of more than 6lg (CFU/mL) of *P.aeruginosa* cells was measured only after a preincubation in PBS, at 10 and 100µM of THPTS. In contrast, the photosensitizing activity of THPTS towards *P.aeruginosa* was definitely blocked in serum.

The ability of THPTS to photoinactivate MSSA, MRSA and Gram-negative *E.coli* bacteria, even in the presence of blood proteins and without any additives which increase membrane permeability, represents an original finding. In a recent paper Maisch and collaborator described the effects of novels XF porphyrin derivates, that kills MRSA at the same (10µM) concentration as THPTS, but unfortunately the author did not test the influence of serum on the photokilling of Gram-negative bacteria using their photosensitizer. More recently they used their compound in an *ex-vivo* model, confirming the bactericidal effect of XF porphyrins [34]. The activation range between 380 to 480nm allowed only a limited penetration (<1mm), thus compatible only with very superficial infection treatment, rarely seen in case of nosocomial infection following surgery. Even if it needed to be use in the 10-100µM range, our new THPTS compound can still mediate an efficient bactericidal function in presence of large amounts of serum; a model much closer to the *in vivo* reality of skin infection. In addition, our compound might be used in a therapeutic alternative against *P aeruginosa* infection, 10µM of THPTS incubated for three hours is sufficient to get rid of all bacterial colonies. Compared to other porphyrin derivates [35] THPTS exhibit higher efficiency against this bacterial strain. THPTS could therefore be use to treat with PDT two major bacterial strain (*S.aureaus* and *P.aeruginosa*) found in multiresistant bacterial infection of skin, lung and blood [36]–[39].

Our results have clearly demonstrated that meso-substituted tetracationic THPTS is an efficient photosensitizer for killing Gram-positive *S.aureus* (MSSA and MRSA) and Gram-negative bacteria *E.coli* and *P.aeruginosa* with infrared light illumination at 760nm. The near infrared wavelength activation leads to deeper penetration of the laser beam, allowing it to stimulate our photosensitizer in thicker tissues (up to 10mm). This is of great interest for the treatment of resistant bacterial infection following surgical intervention. Previous publications described meso-substituted cationic porphyrin that were stimulated in the visible wavelength [25]–[29], [34], leading to a limited penetration (<1mm) depth and much lower efficiency of the PDT. Therefore, even if THPTS is acting at higher concentration than these porphyrins, it appears much more adapted to clinical applications in the treatment of wound infection.

### Mechanism of action and serum influence

Present results together with previously published studies [25]–[29] indicate that positively charged porphyrins are interesting sensitizers for photoinactivation of both Gram-positive and Gram-negative bacteria. The selective mechanism of entry of cationic photosensitizers in bacteria is still poorly understood, but positively charged photosensitizers seem to move across the outer membrane via a self-promoted uptake pathway, in a mechanism involving interaction between divalent cations of the compound with adjacent bacterial lipopolysaccharide [24]. THPTS may therefore cross the outer membrane via such a pathway.

A similar mechanism of entry has also been described for cationic antimicrobial peptide in *P.aeruginosa* [40]. Serum proteins might therefore affect THPTS effectiveness by modifying the lipopolysaccharidic environment present on the bacterial membrane (glycosylation status, charge), and/or bacteria permeability, thus inducing change in the affinity with cationic THPTS. This hypothesis is consistent with the fact that serum had a more drastic effect in Gram-negative bacteria, which possess a more complex membrane structure than Gram-positive bacteria, with a simplified membrane. In those bacteria, we established that the effect of serum can be overcome by modulating either THPTS concentration or incubation time (Fig. 2B and 3B).

### Conclusion

The worldwide increase in antibiotic resistance among different classes of Gram-positive and Gram-negative bacteria has led to a search for alternative antimicrobial therapies, like antimicrobial PDT. At this time, there is no routine application of antimicrobial PDT in the treatment of localized infections. Next step will be to test THPTS effectiveness in vivo, using animal model of bacterial infection. Other groups have developed such model for PDT applications. Hamblin et al., (2003) were able to obtain a high survival rate of 90% in mice infected with P. aeruginosa by using poly-L-lysine (pL)-chlorin e6, whereas all mouse from the untreated control group died rapidly [41]. Zolfaghari et al., (2009) observed a strong reduction of viable S. aureus in their mouse model using methylene blue as a photosensitizer [42]. However, in both studies, photosensitizers did not lead to a complete photokilling of bacteria in vivo compared to in vitro results. Efficiency of above photosensitizers was reduce in vivo due to their inherent limitations: charge, amount of energy needed, excitation wavelength and light penetration, low specificity of the photosensitizer for bacteria vs host cells, effect of serum. Therefore, in vitro results presented here are now calling for further experiments in vivo that may confirm that THPTS can meet all requirements in order to use PDT as an alternative option to antibiotic treatments in clinical practice.

## Materials and Methods

### Bacteria strains

The organisms used in our studies were three typical members of the microflora in wounds: The Gram-positive bacterium *Staphylococcus.aureus* and the Gram-negative bacteria *Escherichia.coli* and *Pseudomonas aeruginosa*. We used the following strains: *S.aureus DSM1104 (ATCC 25923)*, the MRSA strain *S.aureus DSM11729 (ATCC 33592)*, *E.coli DSM 8698* and *P.aeruginosa DSM1117 (ATCC 27853)*.

The bacterial cells were grown aerobically overnight at 37°C in Tryptic Soy Broth (Merck KGaA Darmstadt, Germany). Cells were harvested by centrifugation and suspended in sterile phosphate-buffered saline or sterile PBS supplemented with 10% sterile human serum (HS). The final OD (optical density) of the bacterial suspensions at 600nm, 1cm was 0.015 in all cases. The bacterial suspensions were placed into sterile black well plates with clear bottoms (Costar 3603, Corning Inc., USA).

### Photosensitizer

The novel photosensitizer tetrahydroporphyrin tetratosylat (THPTS), C_72_H_70_N_8_O_12_S_4_, MW 1367.66 was kindly donated by TetraPDT Inc., D-04519 Rackwitz, Germany. THPTS is a highly pure (>99,9% HPLC), water soluble, chemical stable, positively charged compound absorbing with an extinction coefficient of ε = 105,000 M^−1^cm^−1^ at 760,5nm in water. Photosensitizer stock solution (2mM) was diluted in H_2_O and kept in the dark at 4°C. It was further diluted in PBS (pH 7.4) with RPMI 1640 medium without phenol red and supplemented with 10% FCS.

### Photodynamic inactivation of bacteria suspensions

Suspensions of bacteria were exposed to light from a diode laser with a wavelength of 761±3nm (Ceralas D, Ceramoptec GmbH, Bonn, Germany) after incubation with the photosensitizer THPTS for 30, 90 and 180min in the dark at room temperature. The individual wells of the plate were illuminated via an optical fiber from the bottom of the plate. The fluence rate for this setting was about 1W/cm^2^ (measured with Optometer P-9710, Gigahertz-Optik GmbH, Puchheim, Germany). For the used illumination time (100sec) the resulting total light dose was about 100J/cm^2^.

The control samples for dark toxicity were only exposed to the photosensitizer (final concentration of 100µM) without any illumination. After illumination the samples were removed from the wells of the plate, diluted with Tryptic Soy Broth and plated by using spiral plater Eddy Jet (iul Instruments, Barcelona, Spain) on Tryptic Soy agar plates. The numbers of colony-forming units (CFU/mL) were counted after adequate incubation using colony counter Countermat Flash (iul Instruments, Barcelona, Spain).

### Data analysis and statistics

Each experiment was performed at least in triplicate. All primary data is presented as means with standard deviations of the mean. Differences were tested for statistical significance by Student's t test. Probability values less than 5% were considered significant.

### Viability of bacteria

THPTS was added at final concentration of 250µM to a bacterial suspension of *S.aureus* DSM1104 and *E.coli* DSM8698 (OD = 1.5; 600nm; 1cm for both strains. After an incubation time of 90min the suspensions were illuminated with laser light (761nm, 150J/cm^2^). Right after illumination 50µl of the bacterial suspension was mixed with 1µl of LIVE/DEAD *Bac*Light solution (invitrogen, Cat. No. L7012) consisting of a 1∶1-mix of Syto9 and propidium iodide. A live dead assay was then incubated for 15min in the dark before microscopic procedure using Zeiss “Axiovert S 100” and filter set #09 (Carl Zeiss AG, Jena, Germany). Micrographs were taken using a “SPOT slider - RT Realtime” camera system in combination with “SPOT Advanced” software (Visitron Systems GmbH, Puchheim, Germany). Living bacteria were labelled in green using Syto9 nuclear stain, whereas damaged bacteria were labelled yellow as they were also stained in red with propidium iodide. A control was performed by omitting the 760nm illumination in order to test the THPTS toxicity.

## Supporting Information

Figure S1 Effect of high serum concentrations. Photoinactivation of *S.aureus* (MSSA strain: DSM1104) by THPTS after an incubation time of 90min in solutions containing 0 to 100% HS (human serum). Viability of MSSA was determined by a CFU assay. Even in presence of 25% serum, 10µM of THPTS have a dramatic bactericidal effect leading to the total absence of colony. Using 100 µM of THPTS we observed a bactericidal effect of more than 2log even with higher concentration of serum (50 to 100%). Each point is the mean ± standard deviation of three experiments (P<0.005). Control: no THPTS incubation; dark toxicity: THPTS incubation but no light exposure; illumination: photoinactivation using indicated THPTS concentrations. Arrows indicate a complete absence of bacteria.
(0.03 MB DOC)
