# Supplementary material for: Efficient Photodynamic Therapy against Gram-Positive and Gram-Negative Bacteria Using THPTS, a Cationic Photosensitizer Excited by Infrared Wavelength
Source: PLoS One. 2010 Jul 20;5(7):e11674. doi: 10.1371/journal.pone.0011674 (PMC2907405; doi:10.1371/journal.pone.0011674)
Supplement: Supplementary file 1 [file pone.0011674.s001.doc]

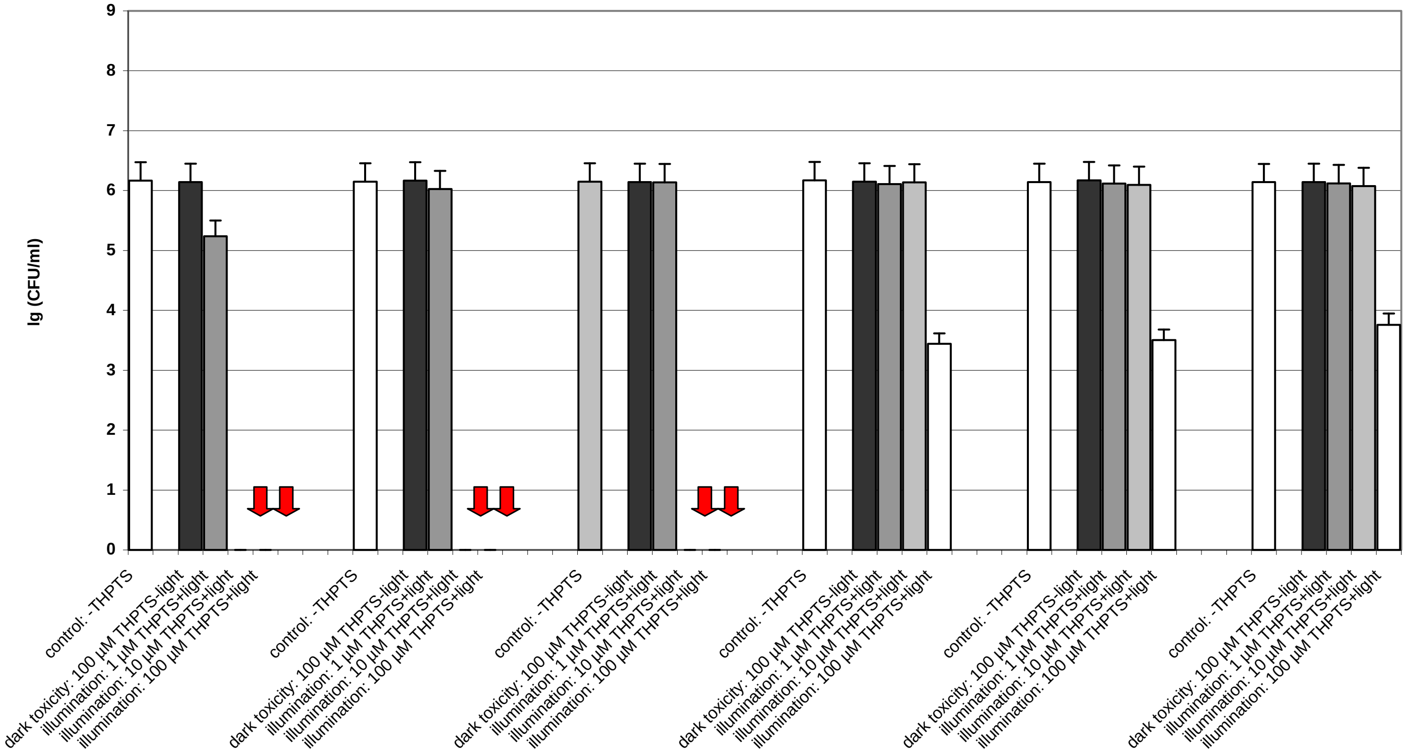

lg (CFU/ml)
9
8
7
6
5
4
3
2
1
0
control: -THPTS
dark toxicity: 100 µM THPTS-light
illumination: 1 µM THPTS+light
illumination: 10 µM THPTS+light
illumination: 100 µM THPTS+light
